# Decoupling of evolutionary changes in transcription factor binding and gene expression in mammals

**DOI:** 10.1101/gr.177840.114

**Published:** 2015-02

**Authors:** Emily S. Wong, David Thybert, Bianca M. Schmitt, Klara Stefflova, Duncan T. Odom, Paul Flicek

**Affiliations:** 1 European Molecular Biology Laboratory, European Bioinformatics Institute, Wellcome Trust Genome Campus, Hinxton, Cambridge, CB10 1SD, United Kingdom;; 2 University of Cambridge, Cancer Research UK - Cambridge Institute, Li Ka Shing Centre, Cambridge, CB2 0RE, United Kingdom;; 3 Wellcome Trust Sanger Institute, Wellcome Trust Genome Campus, Hinxton, Cambridge, CB10 1SA, United Kingdom

## Abstract

To understand the evolutionary dynamics between transcription factor (TF) binding and gene expression in mammals, we compared transcriptional output and the binding intensities for three tissue-specific TFs in livers from four closely related mouse species. For each transcription factor, TF-dependent genes and the TF binding sites most likely to influence mRNA expression were identified by comparing mRNA expression levels between wild-type and TF knockout mice. Independent evolution was observed genome-wide between the rate of change in TF binding and the rate of change in mRNA expression across taxa, with the exception of a small number of TF-dependent genes. We also found that binding intensities are preferentially conserved near genes whose expression is dependent on the TF, and the conservation is shared among binding peaks in close proximity to each other near the TSS. Expression of TF-dependent genes typically showed an increased sensitivity to changes in binding levels as measured by mRNA abundance. Taken together, these results highlight a significant tolerance to evolutionary changes in TF binding intensity in mammalian transcriptional networks and suggest that some TF-dependent genes may be largely regulated by a single TF across evolution.

A multitude of *cis*-regulatory elements, including promoters, enhancers, silencers and insulators, control the initiation and regulation of gene expression. The action of transcription factors (TFs) in recognizing and dynamically binding to degenerate sequence motifs located at regulatory elements plays a key role in transcription. Binding of multiple TFs in close vicinity of one another defines *cis*-regulatory modules that can specify distinct cell fates, and variable occupancy levels of the same *cis*-regulatory module can regulate the same set of target genes to changed biological outcomes (MacArthur et al. 2009). The importance of such cooperative regulation is highlighted by the codependence of TF binding observed across evolution (He et al. 2011; Paris et al. 2013; Stefflova et al. 2013).

Much of our current understanding of the regulatory landscape in animals and the complex transcriptional pathways they control is derived from studies in *Drosophila* and yeast (Biggin 2011). However, mammalian genomes are more vulnerable to random genetic drift due to smaller effective population sizes (Lynch 2007). How such differences have shaped the transcription factor binding landscape and how this then impacts the elaborate gene networks they control is poorly understood. TF binding may evolve under less selective pressure in mammals compared with nonvertebrate species due to the aforementioned population effect. Indeed, a recent study by Cusanovich et al. (2014) showed that following the siRNA knockdown of 56 TFs in a human lymphoblastoid cell line, only a modest change in genome-wide gene expression levels could be detected (∼10% median effect size). Their results also suggest that the majority of binding events have little to no impact on gene expression.

Chromatin immunoprecipitation followed by deep sequencing (ChIP-seq) is widely used to identify in vivo TF binding sites across the genome—producing a quantitative measure at each genomic locus where the TF is bound to DNA. Known as binding intensity or binding occupancy, this signal correlates with sequence motif conservation and also reflects in vivo binding strength (Sun et al. 2013).

Comparative ChIP-seq studies across closely related species have revealed that both TF peak turnover and peak intensity are highly variable in mammals (Schmidt et al. 2010; Stefflova et al. 2013). In contrast, the locations of TF binding sites are generally conserved among yeast and *Drosophila* species (Borneman et al. 2007; Bradley et al. 2010), where ∼50% of binding peaks are shared across *Drosophila* species despite changes to binding intensity levels (Bradley et al. 2010; He et al. 2011).

To examine the coevolution of TF binding and gene expression in mammals, we leveraged liver ChIP-seq data sets for three liver-specific transcription factors, FOXA1, CEBPA, and HNF4A, across four closely related species of mice (Stefflova et al. 2013) and supplemented these data sets by sequencing matching liver transcriptomes for the same mouse species. Given the rapid rate of biochemical change in transcription factor binding, the short evolutionary timescale simultaneously allows adequate time for transcriptional changes to accrue and yet provides sufficient levels of regulatory conservation across species for comparative study. We tracked the evolutionary trajectory of mRNA output over evolutionary time to study its pattern of divergence as well as its covariance with lineage-specific transcription factor binding. Finally, we exploited the availability of genetically modified TF knockout mice to identify the *cis*-binding events that are likely to influence transcriptional output. Analysis of the binding events associated with these TF-dependent genes allowed us to assess the relative contribution of binding intensity change to gene expression across evolution.

## Results

### Gene expression levels are more evolutionarily conserved than collective TF binding

We sought to directly compare the conservation of gene expression levels with that observed for TF binding in closely related mouse species (Stefflova et al. 2013), and thus we generated RNA-seq libraries of liver samples from at least three individuals from the four mouse species used in the prior study: *Mus musculus musculu*s (BL6), *Mus musculus canstaneus* (CAST), *Mus spretus* (SPRET), and *Mus caroli* (CAR) (see Methods). To define orthologous genes across species, we aligned *Mus musculus* cDNA sequences against the genomes of CAST, SPRET, and CAR (Keane et al. 2011; Stefflova et al. 2013). RNA-seq reads were then mapped to their respective genomes, and the expression level for each gene was determined by allocating aligned reads to gene annotations for each species (see Methods; Supplemental Fig. S1). A total of 10,115 putative orthologous genes were expressed across species above a read count threshold of 10. In total, 4465 genes (44% of expressed genes) were differentially expressed in at least one pairwise species comparison (FDR < 0.01). The numbers of differentially expressed genes between species correlated with evolutionary distance and ranged from 705 genes (BL6 versus CAST) to 2686 genes (CAST versus CAR).

**Figure 1.**
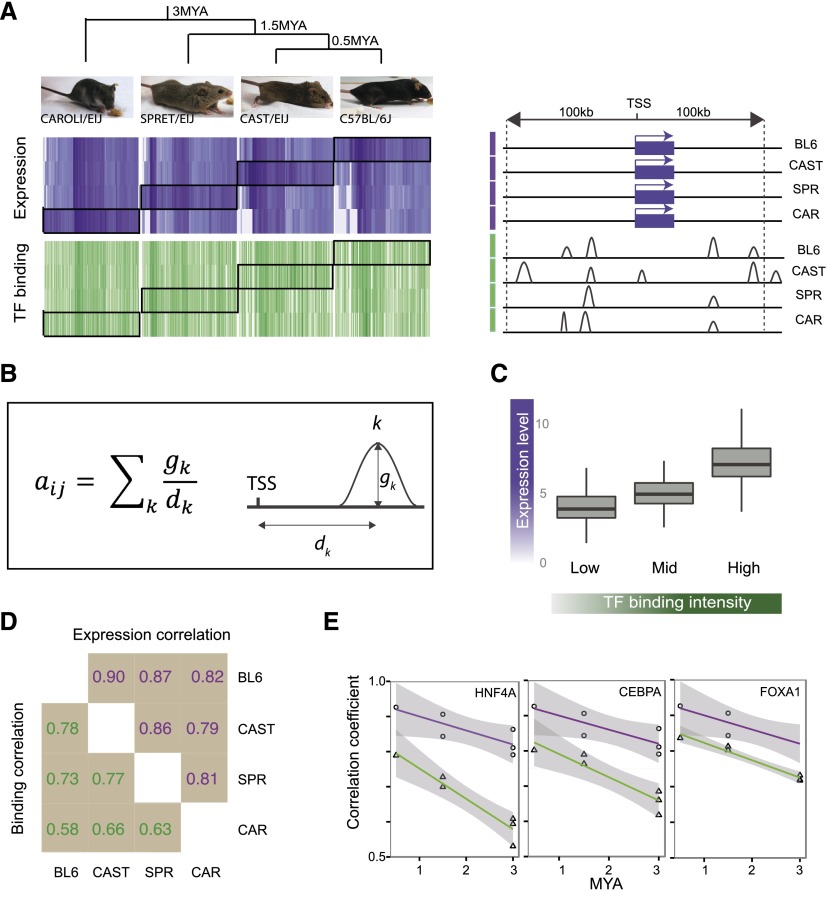
Evolution of transcription factor binding and gene expression between closely related mammals. (*A*) Overview of lineage-specific relationship of TF binding (green) and gene expression (purple), sorted by gene expression. Darker regions in the heatmap denote higher values of binding and expression. Genes were selected for the heatmap based on differential expression in one lineage versus the others with expression values in other lineages also shown. Corresponding binding values in the proximity of each gene are displayed and illustrate the noisy relationship between binding and expression. (*B*) Description of the method used for calculating a binding score for each gene (*i*) for each TF (*j*). Each peak (*k*) was weighted by its distance from any TSS within 100 kb. For each gene, weighted peak intensities for all peaks within 100 kb of either direction from the TSS were summed. (*C*) High expressed genes are more highly occupied by TFs. (*D*) Spearman’s rho for pairwise correlation of expression and binding between mouse species. (*E*) Decay rate of correlation coefficient for binding and expression. Shaded areas represent point-wise 95% confidence intervals.

In order to directly compare TF binding with gene expression, we calculated an integrated binding score representative of the proximal binding intensity of each TF for each expressed gene. Our analysis of TF binding data thus focused on two quantitative measures: (1) Each discrete binding site has what we call a peak intensity, and (2) the collective binding intensity of all individual peaks near each gene was referred to as binding intensity. To calculate a collective binding intensity score for each gene, we took into account the number of peaks, the peak intensities, and peak distance from the transcription start site (TSS), up to 100 kb in both the 5′ and 3′ directions (see Methods; Fig. 1A,B). Distal peaks were down-weighed as proximal enhancers are generally more predictive of gene expression than distal ones (Andersson et al. 2014). We found that an approach using summed binding intensity values linearly weighted by distance provided a better correlation with mRNA levels compared with unweighted summed peak intensities or simple peak counts (e.g., HNF4A BL6 R^2^: using distance weights = 0.13, summed intensity = 0.089, total peak counts = 0.087). Once binding intensities were obtained, we quantile-normalized these values across species and log-transformed the values. We also mean-centered and set the variance of binding values to 1 on a species-specific basis; mRNA counts were similarly processed (see Methods).

More than 50,000 binding sites were found for each TF in each species. Following the above method of peak aggregation, a median of four to seven binding sites were associated to each gene for each species and TF. The maximum numbers of peaks associated with a gene ranged from 61 for CEBPA and FOXA1 to 72 for HNF4A. Approximately 60% of assigned peaks were associated with more than one liver-expressed protein-coding gene.

We found gene expression to be more highly conserved compared to binding intensities across species (expression Spearman’s rho = 0.79–0.92; binding Spearman’s rho = 0.53–0.78) (Fig. 1D). By mapping the correlation coefficient of pairwise binding intensities and expression values against species divergence times using a linear model, we found a marginally faster rate of correlation decay for binding compared to expression (*P* < 0.05; binding slope = −0.4; expression slope = −0.2) (Fig. 1E). To measure the predictive ability of TF binding on gene expression, we performed multiple linear regression on gene expression level using the binding intensities of all TFs as independent variables. Separate regressions were performed for each species. Consistent with findings in human cell lines and fruit flies (Ouyang et al. 2009; Paris et al. 2013), we found binding intensities to be weakly predictive of gene expression (multiple linear regression, adj. R^2^ = 0.21–0.23, *P* < 2.2 × 10^−16^) (Fig. 1C). For all species, regressions that included interaction terms between TFs were significantly more predictive than those that did not (ANOVA, *P* < 2.2 × 10^−16^). For all species, the three-way interaction regression coefficient was significant, indicative of differential interactions between TFs (multiple linear regression, *P* < 1 × 10^−3^).

Overall, these results demonstrate higher evolutionary conservation for gene expression compared to TF binding in the same mammalian system, and confirm that combinatorial TF binding is positively correlated with transcriptional levels of nearby genes.

### Collective binding intensity near TF-dependent genes are preferentially conserved

**Figure 2.**
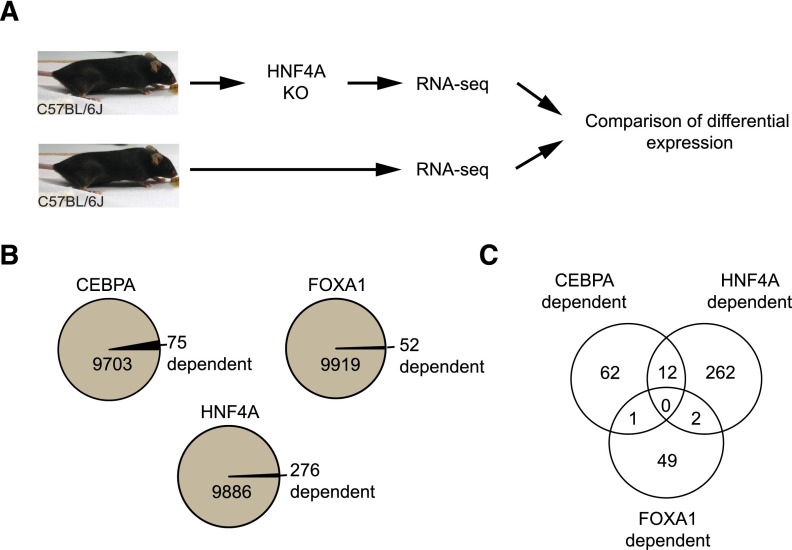
TF-dependent genes are defined by differential expression analyses against wild-type samples after knockout experiments. (*A*) TF-dependent genes are defined as those genes whose gene expression changed in the liver following knockout of the TF. Numbers of HNF4A target genes were conservatively estimated using a stringent *P*-value cutoff. (*B*) TF-dependent genes for CEBPA, FOXA1, and HNF4A respective to total numbers of liver-expressed genes (genes with raw expression counts above 10 reads per species). (*C*) Venn diagram shows the number of common and distinct genes between all TF-dependent gene sets.

To identify protein-coding genes that are most likely to be regulated by our set of TFs, we compared protein-coding mRNA expression in the livers of wild-type (WT) and TF knockout (KO) mice (Fig. 2). We assigned the term “TF dependent” to those genes that show altered expression levels following TF knockout. Conversely, for simplicity, we assigned the term “TF independent” to those genes that did not meet our stringent cutoff for differential expression (see Methods).

Gene expression results for FOXA1 and CEBPA KO mice have been previously reported (Schmidt et al. 2010; Bochkis et al. 2012), and we used these data directly. For HNF4A, we performed RNA-seq using *Hnf4a* KO and WT mice (Fig. 2A). We conservatively estimated that ∼3% of genes showed a clear change in gene expression following *Hfn4a* knockout. TF-dependent protein-coding genes from similar knockout experiments in adult mouse liver for *Cebpa* (Schmidt et al. 2010) and *Foxa1* (Bochkis et al. 2012) comprised of 0.8% and 0.5%, respectively, of the liver-expressed genes in our data set (Fig. 2B).

**Figure 3.**
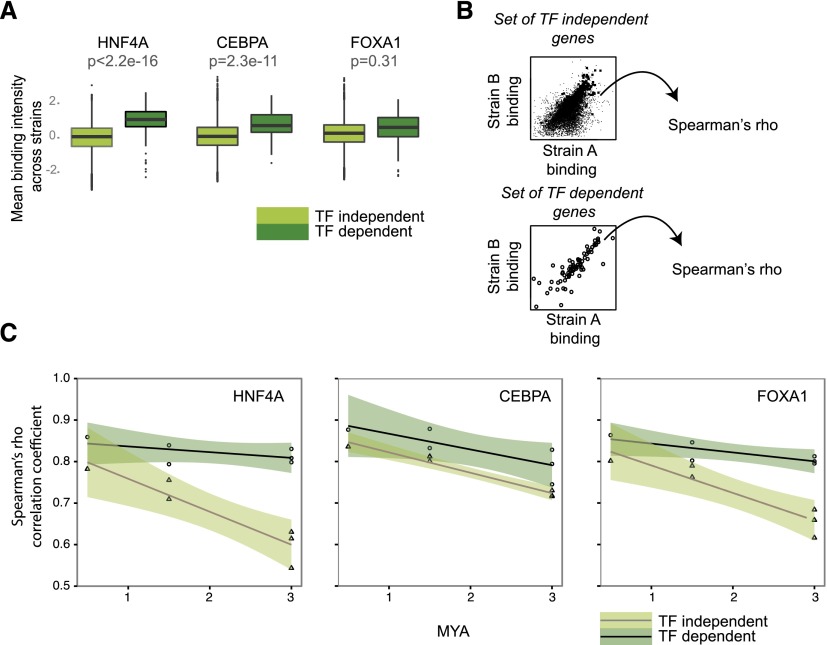
Collective binding intensity is conserved near TF-dependent genes. (*A*) Boxplots comparing collective binding intensities between TF dependent and TF independent for each TF (values are averaged across species). *P*-values were calculated using the Mann-Whitney *U* test. (*B*) Correlation coefficients (Spearman’s rho) were derived from pairwise comparison of collective binding values between taxa for both TF-dependent and TF-independent genes. (*C*) Decay of TF binding correlation coefficient over evolutionary time for the three TFs. TF-dependent genes tend to show greater conservation of collective binding intensity compared to TF-independent genes. Shaded areas represent point-wise 95% confidence intervals.

Compared to TF-independent genes, TF-dependent genes showed a higher abundance of binding sites of the TF near the TSS, suggesting that they are more likely to be directly regulated by the deleted transcription factor; such a model is a general mechanism in fruit flies (Biggin 2011; Paris et al. 2013). Under our stringent significance cutoffs, the genes considered dependent on any of the three TFs were largely distinct from one another (Fig. 2C). TF-dependent genes were also more likely to be differentially expressed between different species, with the exception of FOXA1 (Odds ratio [OR]: HNF4A = 1.4, CEBPA = 5.4, FOXA1 = 0.95). In addition, our data show that mammalian TF-dependent genes typically possessed higher binding intensity compared to genes less sensitive to TF knockdown (Fig. 3A).

We used general linear models to assess whether binding intensities proximal to TF-dependent genes were more conserved. We calculated the correlation coefficients of binding intensities for each pair of species for TF-dependent and TF-independent genes (Fig. 3B,C). The null hypothesis that binding intensities for TF-dependent and TF-independent genes were statistically indistinguishable from one another was tested using the analysis of covariance (ANCOVA) (see Methods). We found that collective binding intensity was better conserved proximal to TF-dependent genes compared to independent genes (ANCOVA; HNF4A: *P* = 4.3 × 10^−4^; CEBPA: *P* = 3.3 × 10^−3^; FOXA1: *P* = 7.5 × 10^−4^) (Fig. 3B,C). Moreover, the rate of binding correlation change over time was slightly, but significantly, faster for HNF4A and FOXA1 independent genes compared to dependent genes (linear regression; HNF4A difference in slope = 0.03, *P* = 8.5 × 10^−3^; FOXA1 difference in slope = 0.02, *P* = 1.9 × 10^−2^).

Average binding intensities across species were negatively correlated with the standard deviation (*P* < 2.2 × 10^−16^, Pearson’s *r* = −0.21 to −0.31) (Supplemental Fig. S2A–C), meaning that sites of strong TF binding show lower variability in TF binding between mouse species. We therefore considered the possibility that this correlation may explain the TF binding stability at target genes.

To explore this, we first restricted the set of TF-independent genes to only those with binding intensities greater than the median intensity of TF-dependent genes. Using a matched cutoff for dependent genes, we measured conservation values, as before, based on pairwise correlation between species, and tested for a difference between the fitted regression lines for restricted TF-dependent versus restricted independent genes. Binding intensities remained significantly less conserved for TF-independent genes for all TFs, suggesting that the conservation of TF binding was not conditional on high binding intensities (ANCOVA; HNF4A: *P* = 4.0 × 10^−7^; CEBPA: *P* = 8.3^−4^; FOXA1: *P* = 9.1 × 10^−3^). In addition, we also subsampled from the set of TF-independent genes to construct a set of TF-independent genes with a similar binding intensity distribution to TF-dependent genes. Comparisons between the TF-dependent and subsampled TF-independent groups were consistent with our previous findings, albeit with reduced statistical significance (HNF4A: *P* = 9.8 × 10^−6^; CEBPA: *P* > 0.1; FOXA1: *P* = 1.8 × 10^−3^) (Supplemental Fig. S3A).

Next, we explored the effect different approaches for integrating peak intensities had to our finding. Intensities of peaks flanking 10 kb on each side of the TSS were used to calculate a binding value for each gene. We found that binding intensities near TF-dependent genes were conserved without distance-based adjustment of peaks, however to a lesser extent (Supplemental Table S1). Significant differences in conservation of binding intensities between TF-dependent versus independent genes were observed for HNF4A and CEBPA (HNF4A: *P* = 2.4 × 10^−4^; CEBPA: *P* = 1.9 × 10^−4^; FOXA1: *P* = 0.7).

We investigated the effect of varying the distance-weighting given to peaks around the TSS by introducing an exponential function that gives greater weight to peaks further away (Supplemental Fig. S4). A constant value, *d_0_*, was used to control the rate at which the intensity of a peak decayed as the distance from the TSS increases (see Methods). A small *d_0_* value will increase the speed of decay, and lessen the contribution of more distant peaks. Because the linear method strongly down-weighted distant peaks, we parameterized the exponential using *d_0_* = 500 and *d_0_* = 5000, both of which increased the contribution of distant peaks (Supplemental Fig. S4). For both exponential parameters, we found similar results to the linear weighting, all of which were substantially superior to no distance-based weighting of TF binding (Supplemental Table S1), supporting the relative importance of proximal TF binding sites in predicting mRNA levels.

In summary, we find that binding intensities close to TF-dependent genes are better conserved than bulk genes. This trend is significant even after taking into account the differences in binding intensity levels between the groups and different strategies of peak assignment to genes.

### Peak intensity, peak count, and proximity of peaks to the TSS are associated with binding conservation near TF-dependent genes

In light of the variable nature of TF peak conservation in mammals, we sought to determine the genomic characteristics of TF binding sites associated with the observed increase in binding intensity conservation near TF-dependent genes. We first investigated the 10 kb upstream of and downstream from each transcription start site as a single regulatory region for both TF-dependent and -independent genes. We compared changes to pairwise correlation values between species to evaluate the contribution made to the conservation of collective binding intensity by (1) the total number of peaks in each proximal promoter; (2) the summed binding; and (3) the average binding intensity within each proximal promoter (summed binding divided by the total number of peaks).

To closely examine the relationship between TF binding conservation and distance from the TSS, we also divided the region surrounding each TSS into 1-kb bins. We then compared the average correlation coefficients of TF binding for TF-dependent and TF-independent genes between the three pairs of mouse species that are of equal divergence times, namely BL6 and CAR, CAST and CAR, and SPRET and CAR.

**Figure 4.**
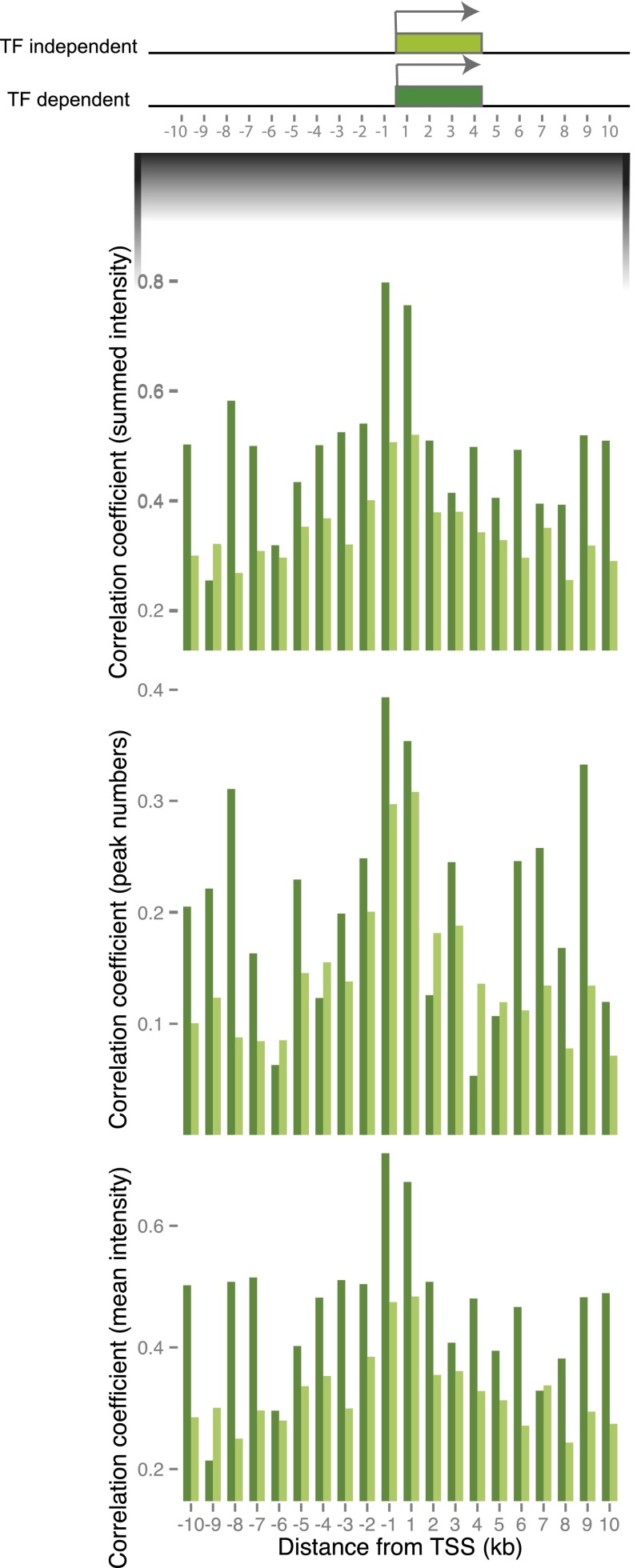
Both peak intensity and total number of peaks are conserved near TF-dependent genes. Spearman’s rho correlation coefficient of summed binding intensities (summation of all peak intensities within binned region), peak counts, and average peak intensities (summed peak intensities divided by the number of peaks in each bin) averaged over BL6 and CAR, CAST and CAR, and SPRET and CAR comparisons. These values are summarized for 1-kb binned distances from TSS for TF-dependent versus TF-independent genes for all three TFs. HNF4A dependent (dark green) and HNF4A independent (light green). For other TFs, see Supplemental Figure S5.

For HNF4A and FOXA1, but not CEBPA, TF-dependent genes showed elevated conservation in summed peak intensities compared with nontarget genes for all bins (Fig. 4; Supplemental Fig. S4) (Mann-Whitney *U* test, across all bins comparing TF dependent and independent; HNF4A: *P* = 4.5 × 10^−5^; FOXA1: *P* = 2.1 × 10^−3^; CEBPA: *P* = 0.25). Peak numbers were also best conserved in the immediate vicinity surrounding the TSS for HNF4A- and FOXA1-dependent genes, particularly in the 1-kb region immediately upstream of the TSS (Fig. 4; Supplemental Fig. S4) (one-tailed paired *t*-test at 1-kb upstream of TSS, *P*-value HNF4A: *P* = 6.5 × 10^−4^; FOXA1: *P* = 2.2 × 10^−2^; CEBPA: *P* = 0.96; Mann-Whitney *U* test across all bins; HNF4A: *P* = 2.3 × 10^−2^; FOXA1: *P* = 7.1 × 10^−3^; CEBPA: *P* = 0.68). Peak intensities remained conserved for these TF-dependent genes near the TSS after normalizing for the number of peaks (Mann-Whitney *U* test; HNF4A: *P* = 3.4 × 10^−5^; FOXA1: *P* = 1.9 × 10^−2^; CEBPA: *P* = 0.25).

We sought to determine the evolutionary scenario that gave rise to total peak counts being more conserved in the general vicinity of TF-dependent genes. Was this due to the conservation of individual specific peaks, or was an average peak number maintained in the face of persistent peak turnover? We obtained peak location information from Stefflova et al. (2013), in which peaks from all species have been mapped to BL6 genome coordinates for the purpose of direct comparison. We considered all peaks that lie within a distance of 10 kb of any TSS. For each peak in BL6 and CAR, peak locations were tested for genomic overlap between species. We used Pearson’s χ^2^ tests to assess whether individual peak locations were more conserved near TF-dependent or TF-independent genes for each TF. These comparisons were performed relative to the distribution of TF-independent peaks.

Individual peaks tend to be more conserved for HNF4A-dependent genes. However, there is a trend across all TFs for individual peaks to be more conserved at TF-dependent versus TF-independent genes, although results were only statistically significant (*P* < 1 × 10^−3^) for HNF4A. This was likely due to the smaller numbers of TF-dependent genes for FOXA1 and CEBPA (mean OR across TFs = 1.26) (Supplemental Table S2). To ascertain whether our results were due to our choice of species for comparison, we performed the same tests between two different species, CAST and SPRET. Consistent with our previous results, we found that with the general exception of HNF4A-dependent genes, peaks were not statistically significantly more conserved for TF-dependent versus TF-independent genes (Supplemental Table S3).

We then examined peak conservation on a gene-by-gene basis by looking at the fraction of conserved peaks per gene (taken here as overlapping locations between BL6 and CAR) for TF-dependent and -independent genes. We assumed a parsimonious model of evolutionary change, which does not take into account peak rebirth, but is likely valid given the brief divergence time between species. We found that a higher proportion of peaks were invariant in the vicinity of HNF4A-dependent genes (Mann-Whitney *U* test; BL6: *P* < 2.2 × 10^−16^; CAR: *P* = 1.4 × 10^−3^). Similarly, we witnessed a similar fraction of invariant peaks in CEBPA and a slightly higher fraction for FOXA1-dependent genes compared to background values (Mann-Whitney *U* test; CEBPA BL6: *P* = 0.82, CAR: *P* = 0.97; FOXA1 BL6: *P* = 6.7 × 10^−3^; CAR: *P* = 3.5 × 10^−2^).

In summary, peaks near TF-dependent genes (HNF4A, FOXA1) were generally more likely to be invariant across species, suggesting they are under selective constraint. Both TF binding intensities and TF peak numbers are conserved. Intriguingly, despite overall conservation of CEBPA binding intensities for CEBPA-dependent genes (Supplemental Table S1), the exact locations of peaks near CEBPA-dependent genes were not maintained across species (Supplemental Tables S2, S3). This may suggest that overall binding intensities at a locus are conserved despite the turnover of individual peaks.

### The transcription of genes dependent on HNF4A and CEBPA show increased variability over evolution

One intuitively appealing model is that the transcription of genes required for a tissue’s function, many of which are directly bound by tissue-specific TFs, would be more stable over evolutionary time. To test this, we used the change in correlation coefficient of mRNA levels between species to measure the change in correlation for mRNA abundance over time.

**Figure 5.**
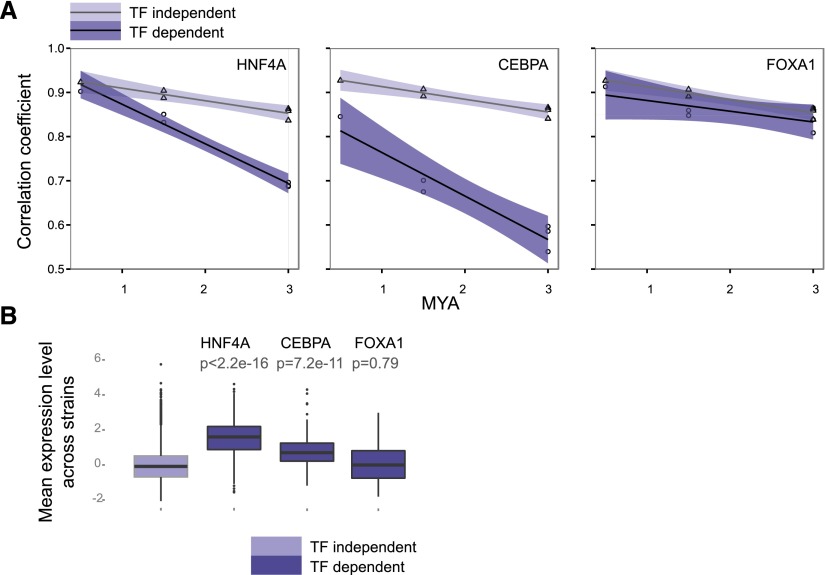
HNF4A and CEBPA TF-dependent genes show divergent transcriptional output. (*A*) Evolutionary change for gene expression for TF-dependent versus TF-independent genes for the three TFs. Darker shading denotes the point-wise 95% confidence interval for TF-dependent genes, whereas lighter shading represents the interval for TF-independent genes. (*B*) Mean gene expression level across species for genes independent of the three TFs and TF-dependent genes for each of the TFs. *P*-values were calculated by comparing TF-dependent versus TF independent gene expression values using the Mann-Whitney *U* test.

In contrast to this intuition, we found that gene expression of TF-dependent genes varies more than bulk genes, even across closely related mouse species; this contrasts sharply with the increased conservation in TF binding found near TF-dependent genes (Fig. 5A). In contrast to what we observed for TF binding (Fig. 3C), expression of TF-dependent genes was less conserved and changes at a relatively faster rate compared with bulk genes. This trend was statistically significant for CEBPA (*P* = 1.7 × 10^−3^) and HNF4A (*P* = 4.7 × 10^−5^), but despite a similar trend, not significant for FOXA1 (*P* = 0.04). A significantly faster rate of change in the level of gene expression conservation was also observed for CEBPA and HNF4A-dependent genes (CEBPA delta slope = 0.03; HNF4A delta slope = 0.03). To explain the coupling of divergent gene expression with tightly conserved binding intensities at TF-dependent genes, we hypothesized that TF-dependent genes were more sensitive to changes in binding, with slight perturbations of regulator occupancy capable of triggering a disproportionate transcriptional response (Figs. 3C, 5A).

To further explore this result, we correlated mean expression levels against their variance across species and found that average expression values across species were slightly negatively correlated with their standard deviation as a result of the log transform (*P* < 2.2 × 10^−16^; Pearson’s *r* = −0.08) (Supplemental Fig. S2D). Thus, the increased variance observed in the more highly expressed TF-dependent genes suggests that our results could not be due to an underlying mean-variance relationship in liver gene expression (Fig. 5B). Applying a variance stabilizing transformation to the count data such that the values were approximately homoscedastic supported this conclusion.

We assessed whether our findings could be due to differences in expression levels between TF-dependent and TF-independent genes by subsampling TF-independent genes to identify a collection with a similar distribution in expression values to the TF-dependent genes. This analysis revealed that TF-dependent genes are more highly variable in gene expression compared to subsampled TF-independent genes with similar expression distributions, consistent with our previous findings (HNF4A: *P* = 6.35 × 10^−6^; CEBPA: *P* = 4.7 × 10^−4^; FOXA1: *P* = 0.01) (Supplemental Fig. S3B). Furthermore, restricting the analysis set to those genes that were expressed at least as highly as the median expression level in the TF-dependent gene set did not change the overall results (HNF4A: *P* = 3.0 × 10^−3^; CEBPA: *P* = 0.01). TF-dependent genes also did not show increased levels of inter-species variability compared to TF-independent genes across taxa and TFs (Kolmogorov-Smirnov one-tail test: HNF4A mean across species *P* = 0.67; CEBPA mean across species *P* = 3.6 × 10^−2^; FOXA1 mean across species *P* = 0.35).

We obtained similar results when we extended our analysis to include BL6 to rat evolutionary comparisons using published rat liver mRNA-seq data (ArrayExpress accession: E-MTAB-867) (Supplemental Table S4; Kutter et al. 2012). Because transcript abundance for the mouse-rat data set was estimated through de novo transcriptome assembly without mapping reads to a genome, the increased divergence in transcriptional variance of TF-dependent genes appears robust to RNA-seq analysis methods.

To summarize, HNF4A- and CEBPA-dependent gene expression was less conserved and changed at a relatively faster rate compared with TF-independent genes. That an increased level of transcriptional variation was found for TF-dependent genes despite an increased conservation of overall binding intensities may at first seem counterintuitive. However, this would indeed be expected if TF-dependent genes were, as a whole, inherently more sensitive to changes in TF intensities, where in contrast, the expression of TF-independent genes was buffered against changes to TF binding levels.

### A Brownian motion model reveals TF binding evolution is typically decoupled from gene expression evolution in mammals

**Figure 6.**
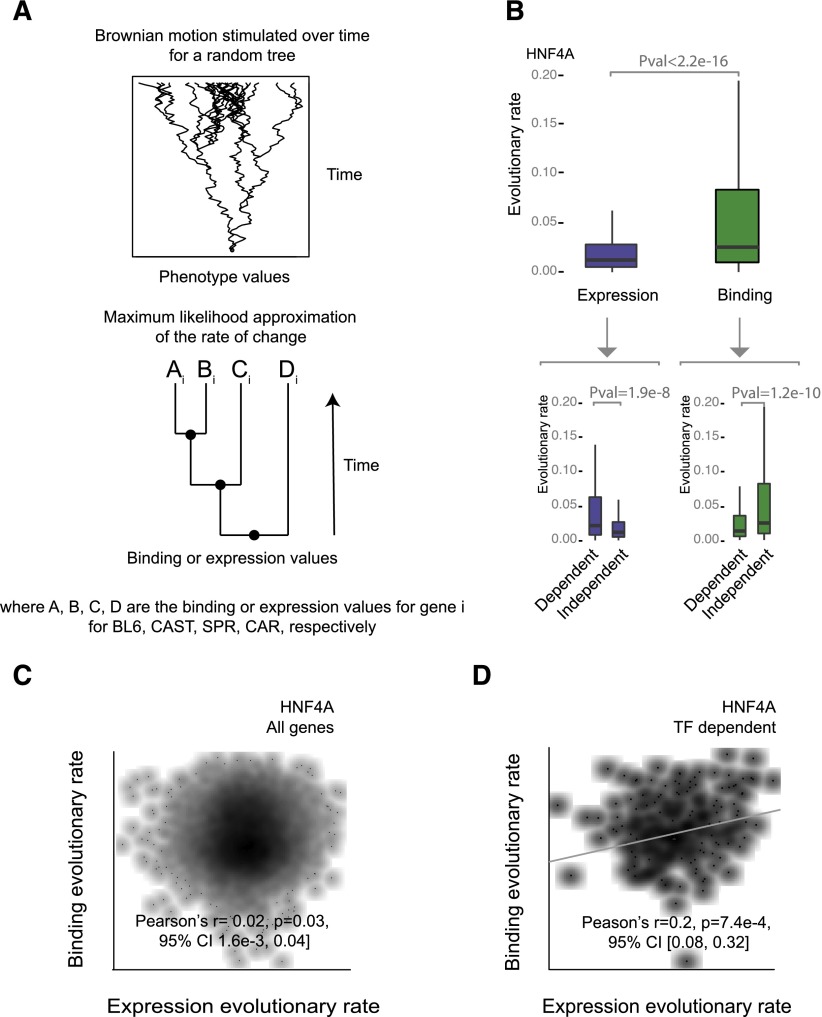
Brownian motion analysis reveals decoupling of TF binding and gene expression evolution rates. (*A*) A phylogenetic generalized least-squares model based on evolution by Brownian motion was used to estimate the evolutionary rate of binding and expression change. The most likely binding and expression rates for each gene were estimated based on the topology of the phylogeny and the binding intensity and expression values for each species. (*B*) Boxplots compare the evolutionary rates of binding and expression for HNF4A. *Lower* boxplots contrast the evolutionary rates for target versus nontarget HNF4A genes separately for binding and expression. (*C*) Density scatterplots showing the rate of HNF4A binding evolutionary change against gene expression for all genes. Correlation was calculated using Pearson’s method with log-transformed values. (*D*) Density scatterplots showing the rate of binding evolutionary change against expression for HNF4A TF-dependent and TF-independent genes. Correlation was calculated using Pearson’s method with log-transformed values.

To test the hypothesis that the transcription of TF-dependent genes was inherently more sensitive to changes in TF binding intensities, we used a Brownian motion model of continuous character change (Felsenstein 1985) to estimate and compare evolutionary rates of binding and expression change. We modeled these traits for all expressed genes by applying a maximum likelihood strategy to fit a rate of binding change and a rate of expression change, which were conditional on the expected phylogeny of the mice species. This analysis was performed for each gene and separately for binding and gene expression (Fig. 6A).

Rates of TF binding change were modestly correlated between TFs (Spearman’s correlation *P* < 2.2 × 10^−16^; FOXA1 versus CEBPA rho = 0.27, FOXA1 versus HNF4A rho = 0.33, CEBPA versus HNF4A rho = 0.34). The rate of binding evolution was, on average, greater than that for gene expression and was also more variable around the mean, consistent with evidence that transcriptional output is better conserved than binding (Fig. 6B). Average expression levels across species also showed a significant, but noisy, negative correlation with gene expression evolutionary rates; in other words, highly expressed genes tend to evolve more slowly (*P* < 2.2 × 10^−16^; Pearson’s *r* = −0.13) (Fig. 6B).

Most notably, across all expressed genes we found no correlation between the rate of collective binding intensity change and the corresponding rate of change in mRNA transcript level at each gene (HNF4A *P* = 0.03, Pearson’s 95% CI [2 × 10^−3^, 4 × 10^−2^]; CEBPA *P* = 7.4 × 10^−3^, Pearson’s 95% CI [7 × 10^−3^, 5 × 10^−2^]; FOXA1 *P* = 0.15, Pearson’s 95% CI [−5 × 10^−3^, 3 × 10^−2^]) (Fig. 6C).

This result is in clear contrast to the significant, albeit noisy, correlation found between expression and nearby TF binding within single eukaryotic species (Ouyang et al. 2009; Paris et al. 2013; Andersson et al. 2014). That the rate of binding and expression change is not correlated despite correlation between gene expression and TF binding suggests that tight regulation of the precise binding level of these liver-specific TFs is not required for stable gene expression in adult liver. Similarly, an independent relationship between binding variance and expression variance has been reported in *Drosophila* embryos (Paris et al. 2013).

Additionally, we fitted the same data sets to the more complex Ornstein-Uhlenbeck model, which adds two parameters to the Brownian motion model to estimate selection, stabilized to an optimal binding or expression value. Likelihood tests supported the simpler Brownian motion model over the more complex one. This was expected given our small sample sizes with large numbers of multiple *P*-value correction tests (Holm method) due to the large number of expressed genes.

Hence, we do not observe covariance of binding and expression evolutionary rates for the majority of expressed genes over time. These results suggest that overall gene expression levels are modulated in liver largely independently of variations in binding intensities of these liver transcriptional regulators across species time.

### HNF4A-dependent genes show subtle covariance in transcriptional and binding rates of evolutionary change

Given that TF-dependent genes appeared more transcriptionally sensitive to TF binding, we hypothesized that TF-dependent genes may show covariance in their rates of binding and transcriptional change, despite the little effect observed for bulk genes. We thus compared the evolutionary rates for binding and expression for genes dependent on HNF4A as a representative TF and found a significant linear correlation between rates of binding and expression change (*P* = 7.4 × 10^−4^, Pearson’s 95% CI [0.08, 0.32], permutation test *P* < 1 × 10^−4^) (Fig. 6D). In contrast, HNF4A independent genes did not show a linear relationship (*P* = 0.03, Pearson’s 95% CI [3 × 10^−3^, 0.04]). However, similar correlations were not found for CEBPA- or FOXA1-dependent genes. This could reflect either functional differences between the TFs, or qualitative differences in the gene expression data sets from the various species of genetically engineered mice. Notably, HNF4A-dependent genes were also typically more highly expressed and possessed greater numbers of binding sites specific to the TF compared with CEBPA- and FOXA1-dependent genes (Fig. 5A).

In summary, although transcriptional rates of change are largely independent of variance in binding intensity across bulk genes, a subtle but significant linear relationship exists for HNF4A-dependent genes. Taken together, our results suggest that TF-dependent genes are more sensitive to variations in binding intensity and also imply that TF-dependent genes are more likely to be regulated by the action of a single TF.

### Effects of cooperativity of binding intensities among TFs

Given that cooperativity between transcription factors is known to increase the explanatory power of regulatory binding to gene expression in a single species, we used a multiple linear regression to test whether the collective rates of change in HNF4A, FOXA1, and CEBPA binding intensities could explain the evolutionary rate of change in mRNA levels. After taking into account the evolutionary rate of all three TFs, no significant correlation was found between the rates of binding and expression change (adj. R^2^ = 6.7 × 10^−4^, *P* = 0.02, R regression formula: gene expression rate of change ∼ HNF4A rate of binding change + FOXA1 rate of binding change + CEBPA rate of binding change).

We further tested whether the rate of change in TF binding intensity is correlated to binding intensity levels, i.e., are genes of higher binding intensities associated with faster rates of binding change over evolutionary time? Binding intensities of different TFs were not strongly predictive of the rate of binding intensity evolution (adj. R^2^ = 0.08 to 0.16, *P* < 2.2 × 10^−16^, multiple linear regression where binding rate of change for HNF4A was predicted by the binding intensities of all TFs). Significant interactions between HNF4A and FOXA1 binding were positively correlated with the rate of binding evolution (*P* = 5.1 × 10^−9^ to 8.0 × 10^−3^, multiple linear regression). Indeed, cobinding of HNF4A and FOXA1 occurs more frequently than other TF pairings among the three TFs (Stefflova et al. 2013). Except in BL6, we also found a significant positive three-way interaction between all TFs in predicting the rate of binding change. The marginal coefficients of independent variables were negative for all species, indicating that in the absence of CEBPA and FOXA1 binding, the rate of HNF4A binding change was slower. These results together indicate that faster rates of binding change are loosely associated with both higher binding intensities and interaction (statistical) among cobinding TFs.

We also tested whether genes of high binding intensities were associated with an increased rate of gene expression change. For each species, we used multiple linear regression to predict the rate of expression change using the binding intensity of HNF4A, CEBPA, and FOXA1 and their interactions terms. TF binding intensities for each species do not appear to account for rates of gene expression change despite a significant statistical effect (multiple linear regression, adj. R^2^ = 7.5 × 10^−3^ to 0.01, *P* < 2.2 × 10^−16^ to 8.6 × 10^−15^). However, interaction of binding intensities between both FOXA1 and CEBPA and between FOXA1 and HNF4A were consistently predictive of expression evolutionary rate in all taxa, albeit with negligible effect sizes (multiple linear regression, *P* = 3.3 × 10^−5^ to 0.03). Both effects were positively associated with changes in expression rate.

## Discussion

To explore the evolutionary relationship between TF binding and gene expression for three liver-specific TFs, we generated novel transcriptome data to combine with matched TF binding maps in four closely related mouse species. We developed an integrated analysis of peaks near protein-coding genes, which allowed us to compare TF binding intensities with gene-specific transcriptional responses across evolution. Transcript levels between wild-type and transcription factor knockout mice were compared to identify those genes whose stable expression was reliant on the transcription of the deleted TF. Given the pervasive and often functionally neutral nature of TF binding (Biggin 2011), our approach defined the subset of genes where TF binding was required.

Our analyses newly reveal an unexpected relationship between mammalian tissue-specific regulatory programs and gene expression divergence. We show, at high resolution using an integrated analysis exploiting TF knockout mice, that transcription factor binding intensity and magnitude of gene expression are largely decoupled in mammalian tissues. This effect exists despite the preferential conservation of average TF binding intensities and peak numbers near the TSS of genes transcriptionally dependent on the factor.

In contrast to findings in other species (Biggin 2011; Paris et al. 2013), we detected a modest correlation between HNF4A binding and expression levels. This suggests that certain genes may be predominately modulated by a single TF. Variable buffering in transcriptional responses may have significant phenotypic consequences for evolutionary adaptation as well as disease phenotypes.

Although both CEBPA- and FOXA1-dependent genes did not show correlation in evolutionary rates of binding and expression change, binding intensities near these genes were preferentially conserved. This suggests that binding intensity may be conserved at these genes for a functional role that is uncoupled from the rate of mRNA production. Indeed, a number of lines of evidence suggest the different functional roles of the TFs in this study may account for the differences in results observed between the TFs. For example, HNF4A-dependent genes also tended to be more highly expressed and were located proximal to a greater number of binding sites than CEBPA- and FOXA1-dependent genes. We also found that certain groups of target genes (CEBPA and HNF4A) were associated with a more variable transcriptional profile across evolution. In contrast, the transcriptional output of FOXA1-dependent genes did not appear to deviate significantly from bulk genes across species. FOXA1 acts as a pioneer factor without which other TFs may not bind, establishing competence for gene expression (Sérandour et al. 2011). Its binding has also been reported to have a “bookmarking” effect during mitosis in liver cells (Caravaca et al. 2013). Hence, FOXA1 may regulate genes in a manner that is largely transcriptionally independent of mRNA abundance. Additionally, that full deletion of HNF4A and FOXA1, but not CEBPA, are embryonically lethal further suggests that functional differences between TFs may significantly contribute to the differences observed between TFs in our study (Bernardo and Keri 2012; Bonzo et al. 2012). However, it is also possible that our evolutionary models were not sensitive enough to explain the rates of change. Although the true evolutionary scenario is likely to be more complex than the Brownian motion model used here, given short divergence times, the lack of obvious differences in liver physiology and function between species, and the limited numbers of species for comparison, a simple model of evolutionary drift was deemed most appropriate and was found to be more suitable than a complex model invoking stabilizing selection.

That little correlation exists between evolutionary changes in TF binding intensities and gene expression appears to contradict the observed (adj. R^2^ ∼ 0.2 from this study) and widely reported (Ouyang et al. 2009; Paris et al. 2013) correlations between expression and binding intensities within a single species. However, several potential and not mutually exclusive reasons may explain this disconnect. First, if the majority of binding sites do have little influence on transcriptional levels, a substantial amount of evolutionary decoupling would be expected because sites near to most genes would be evolving under genetic drift. Indeed, the small correlation observed for HNF4A-dependent genes supports the idea that the majority of binding sites are evolving neutrally. A second possible explanation for the incongruities between the within- and cross-species observations is the presence of functional redundancies among TFs, whose overlapping roles lead to differential binding combinations and intensities resulting in similar transcriptional effects. Hence, gene expression is buffered or “canalized” across evolution. Such redundancies have been widely reported (Biggin 2011). Indeed, TF binding motif strength in yeast has been found to better correlate with gene expression levels when a biological response is orchestrated by a single TF compared with genes controlled by the actions of multiple TFs (Tirosh et al. 2008).

Although we recognize that TF dependency spans a continuum, we have deliberately demarcated between TF-dependent and TF-independent genes using set cutoffs as a way to determine relative evolutionary conservation. This allowed us to directly compare a distribution of covariance values between the two groups of genes. However, the exact extent of such a continuum on overall tissue function is unknown. Additionally, we cannot discount that observed differences between TF-dependent genes are in part due to experimental design as HNF4A-dependent genes were identified using next-generation sequence data, whereas FOXA1- and CEBPA-dependent genes were defined with array data. Finally, there may be an unseen role of post-transcriptional modifications between species on the overall abundance of mRNA transcripts.

In summary, peak intensities near TF-dependent genes are preferentially conserved in a collective manner. HNF4A-dependent genes proximal to highly occupied binding sites tend to be more transcriptionally sensitive to changes in binding intensities over evolutionary time. Except for a small number of HNF4A-dependent genes, comparison of binding and expression evolutionary rates reveals extreme tolerance of mRNA abundance to binding variability, suggestive of extensive redundancy in TF networks. Variability in the extent of TF binding buffering on transcriptional response may have significant phenotypic implications in both species evolution and human disease.

## Methods

### Species-specific ChIP-seq data

The three TFs chosen for this are heavily investigated, liver- and lineage-specific TFs. All three TFs are of different protein families, representing a cross section of the kinds of proteins and their interactions that control regulatory DNA. We choose to work in liver as it is a relatively homogeneous tissue, comprised of mainly (∼70%–80%) hepatocytes (Si-Tayeb et al. 2010). The TFs chosen are generally well characterized functionally and have mouse genetic knockouts. Additionally, the antibodies for these TFs have been well tested, and we have used this system to model in vivo TF evolution (Schmidt et al. 2010; Stefflova et al. 2013).

Liver ChIP-seq data sets for the four inbred mouse species were generated by Stefflova et al. (2013) (ArrayExpress accession: E-MTAB-1414). The data set was comprised of two biological replicates for each species for three transcription factors (HNF4A, CEBPA, and FOXA1). Binding data were processed with methods identical to those described in Stefflova et al. (2013). Briefly, reads were aligned using BWA (Li and Durbin 2009) with default parameters. Peak locations and intensities were called by SWEMBL (https://github.com/stevenwilder/SWEMBL) using genomic DNA as control. Motif searches at regions directly flanking predicted peak summits were carried out to confirm the presence of expected TF binding motifs. Final peak sets contained peaks called in both biological replicates. Peak intensities were taken as the total number of reads that make up a peak for each set of pooled replicates. To account for differences in sequencing depths between species, distributions of binding intensities were quantile normalized. This was performed separately for each TF.

### Association of binding sites to genes

For each gene, we assigned a binding score that is a function of the number of proximal peaks, peak intensity, and distance from TSS. Peak intensities for all peaks that reside in the region 100 kb upstream of and 100 kb downstream from each TSS were first weighted by dividing its peak intensity by the distance to the respective TSS and then summed as follows:
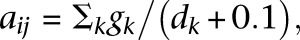
where *g*_*k*_ is the peak intensity of the *k*th peak of the TF *j*; and *d*_*k*_ is the distance of peak *k* to the TSS of gene *i.* This resulted in a single binding value (*a*_*ij*_) per gene for each species. These values were then quantile normalized across species, a pseudocount of 1 × 10^−4^ was added prior to log-transformation. They were also mean-centered, and the variances were set to 1 on a species-specific basis. Again, this does not change the shape of the distribution for these values and was done to compare binding and expression rates across species. We used alternative methods of binding association to assess the degree to which peak turnover and distance to TSS contribute to overall binding changes: (1) We aggregated peaks by summing all binding intensities within the 200-kb region surrounding the TSS (the difference to the method described above is that this did not take into account the distance of individual peaks to the TSS); and (2) we took a binary approach to binding whereby each peak is denoted only by its presence. The binding score was obtained for each gene by counting the number of peaks in the 200-kb window encompassing the TSS.

In order to explore the effect of our peak intensity integration strategy on our findings, we performed the same analysis, comparing binding conservation, using a method that incorporates a parameter controlling the rate of exponential peak decay. The parameter changes the level of contribution a peak makes depending on its distance from the TSS (Ouyang et al. 2009).
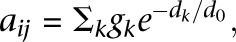
where *g*_*k*_ is the peak intensity of the *k*th peak of the TF *j*; *d*_*k*_ is the distance of peak *k* to the TSS of gene *I*; and *d*_*0*_ is a constant which controls the speed of peak decay. We reanalyzed our data using *d*_*0*_ = 500 and 5000, where a small *d*_*0*_ increased the rate at which peaks decayed relative to distance from the TSS. Compared to our method, both *d*_*0*_ values tested produce less down weighing of distant peaks (Supplemental Fig. S4).

### Species-specific RNA-seq data generation

RNA-seq libraries were prepared for 3–4 biological replicates of perfused liver samples from each of the identical four species from which binding data was obtained. Mice used for RNA-seq analysis were from the same colony and reared under identical conditions as those used for the ChIP-seq study. In total, three biological replicates were produced for each BL6, CAST, and SPRET, and with four replicates sequenced for CAR. BL6 and CAST RNA-seq data sets were previously published in Goncalves et al. (2012) (ArrayExpress accession: E-MTAB-1091).

Wild-type mice were maintained at the University of Cambridge, CRUK (Cambridge Institute under the auspices of a UK Home Office license). The livers were freshly dissected and flash frozen in liquid nitrogen prior to RNA isolation. About 20 mg of each tissue was homogenized in 600 μL RLT buffer (Qiagen) with β-mercaptoethanol using ceramic beads (Precellys). RNA was extracted using RNeasy kit (Qiagen), and DNA was digested using TURBO DNase (Ambion). The quality of the total RNA was assessed by Bioanalyzer Eukaryote Total RNA Nano Series II chip (Agilent). Polyadenylated mRNA was enriched from the total RNA using the PolyATract mRNA isolation system (Promega). Directional double-stranded cDNA was generated according to the method of Parkhomchuk et al. (2009), using the SuperScript Double-Stranded cDNA Synthesis kit (Invitrogen), with uracil substituted for thymine in the second strand. On average, 250 ng of double-stranded cDNA in 300 μL volume was fragmented by sonication using Bioruptor (Diagenode, 30 s on/off, 10 min total sonication time), end repaired, A-tailed, and a sequencing library prepared for the Illumina platform using the Paired End Oligo Only kit (Illumina) according to the manufacturer’s instructions (with the adapters diluted 1:10). Strand specificity was then introduced by digestion of the second strand of cDNA using uracil-N-glycosylase. Each library was PCR-amplified using Illumina’s PE primers, size selected (200–300 bp) performed by 2% agarose gel electrophoresis, followed by paired-end 75-bp sequencing on an Illumina GA IIx according to the manufacturer’s instructions in the Genomics Core facility of the Cambridge Institute.

### Species-specific mRNA abundance quantification

Reads were aligned to species-specific genomes that were constructed by the addition of species-specific SNPs (Keane et al. 2011; Stefflova et al. 2013) to the NCBI37/mm9 assembly of the mouse genome as detailed in Stefflova et al. (2013). To construct gene sets for CAST, SPRET, and CAR, the Ensembl version 72 *M. musculus* gene set (Flicek et al. 2013) was mapped using BLASTN (Altschul et al. 1997) against the respective genomes. Searches were performed using exons derived from the longest *M. musculus* transcripts. Only the best match below an *E*-value cutoff of 1 × 10^−5^ was kept for each exon query. Of 21,783 protein-coding transcripts, 21,061 showed 97% or more conservation in transcript lengths across all species (comparison against BL6).

RNA-seq reads were trimmed using Trimmomatic (Lohse et al. 2012) (leading and trailing bases below a *phred* quality score of 25 were removed, up to a minimum length of 70 bp; LEADING:25; TRAILING:25; MINLEN:70). Reads were then mapped to their respective genome using GSNAP with a maximum of three mismatches and filtered to keep only uniquely mapping reads (Wu and Nacu 2010). Python package HTseq (Anders et al. 2014) was used to obtain counts for each gene in each species by counting reads binned by alignment position to annotated gene locations (-union setting). Exon counts were summed to obtain a count value per gene for each replicate of each species. Differential expression analyses and between replicate library size normalization were performed using DESeq (Anders and Huber 2010). Differential expression analyses were performed in a pairwise species manner using DESeq taking advantage of biological replicates, and additionally with multispecies comparison using a generalized linear model (GLM) implemented in edgeR (Robinson et al. 2010), and estimating dispersion on a gene-wise basis. Both methods produced similar results. For evolutionary analyses, a single expression value for each gene per species was obtained by taking the mean of normalized expression values between replicates. Genes with average read count of 10 or below, corresponding to a transcript per million (TPM) threshold of ∼1–1.5, in any species were removed. Values were then log-transformed and mean-centered, and their variances were set to 1 on a species-specific basis. This does not change the shape of the distribution for these values and was done to allow comparison of binding and expression rates across species. Variance stabilizing transformation was performed using DESeq after a blind estimation of the variance function. Raw counts and normalized values are available in the Supplemental Data file.

### HNF4A KO data generation

Liver-specific HNF4A null mice were generated by inducible CRE-loxP-mediated excision of exons 4 and 5 of the *Hnf4a* gene. To this end, *Hnf4a*^*lox/lox*^ (Hayhurst et al. 2001) mice were crossed with *TTR-Cre ind* (Tannour-Louet et al. 2002) mice. One-month-old *Hnf4a*^*lox/lox*^*/TTRCre ind* mice were intraperitoneally injected with 2 mg/20 g/day of tamoxifen for 5 consecutive days. Analysis was performed at postnatal day 45. The library was prepared in a similar way with the exception of attaching a single end oligo, followed by single-end 36-bp sequencing.

### Definition of TF-dependent genes

The genes that were further sorted into dependent and independent sets had two characteristics: (1) their component genes had to be expressed above a normalized expression value of 10; and (2) a TF binding event was located nearby. After these criteria were met, TF-dependent genes were identified as those genes whose expression was altered following knockout.

To identify HNF4A-dependent genes, RNA-seq reads derived from three liver biological replicates of BL6 HNF4A KO and one BL6 WT mice were quality filtered and aligned against the NCBI37 mouse genome assembly allowing for one mismatch. Multiple mapping reads were removed, and reads were binned into gene annotations, as described above. edgeR was used to normalize between the replicates and test for differential expression between KO and WT samples. Because there was only one WT sample sequenced in the same batch as the KO samples, we also performed differential expression comparisons of KO against the BL6 WT samples described above. Conservatively, we took the intersection of genes with a Benjamini-Hochberg-corrected *P*-value below 1 × 10^−3^ for both analyses as those genes transcriptionally dependent on HNF4A (Benjamini and Hochberg 1995).

CEBPA-dependent genes were defined by Schmidt et al. (2010) using microarray data from BL6 CEBPA knockout mice (ArrayExpress accession: E-MTAB-178). FOXA1-dependent genes were determined using Limma (Smyth 2005) with a Benjamini-Hochberg adjusted *P*-value cutoff of 0.05 on microarray profiles of FOXA1 null and wild-type mice (Benjamini and Hochberg 1995; Bochkis et al. 2012) (ArrayExpress accession: E-MEXP-3426).

### Binding and expression evolution analysis

Spearman’s rho was calculated in a pairwise manner between species. Where mentioned, we produced empirical *P*-values by recalculating the correlation coefficient by resampling the number of TF-dependent genes from those not sensitive to TF KO. *P*-values were estimated after 10,000 rounds of resampling with replacement using the count of the number of times a rho value more extreme than that seen for TF-dependent genes was observed.

Analyses were carried out using general linear models to test the difference between TF-dependent and TF-independent decay in correlation coefficient over evolutionary time. To test whether significant differences existed, for each TF we performed an analysis of covariance (ANCOVA) using the pairwise Spearman’s correlation coefficients between species as a dependent variable against the Boolean value of whether a gene is sensitive to KO and evolutionary time as predictor variables. The formula for defining the ANCOVA model is the following:
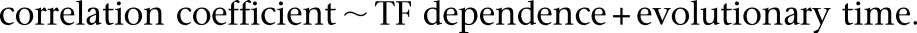


A second model incorporating an interaction effect between TF dependence and evolutionary time was calculated, and we used ANOVA to test between both models in order to assess whether removal of the interaction term significantly affected the fit of the model. If a significant interaction between TF-dependent and bulk genes exists, we fitted separate linear regression models to estimate their respective rates of change. If a significant interaction effect was identified, where *P* < 0.005, the *P*-value reported for that variable is that of the interaction term. Analyses were performed using R (R Core Team 2014). ANCOVA was performed using the “aov” function.

To subsample TF-independent genes to obtain a set of genes with similar binding intensities or expression levels as TF-dependent genes, we identified, for each TF-dependent gene, the TF-independent gene with the most similar mean binding intensity or mean expression value to the binding intensity or expression value for the dependent gene.

Data sets for binning analyses were quantile-normalized and log-transformed prior to analyses. To study the contribution of peak intensity to TF binding conservation, we calculated average binding intensity for each gene for each TF. This metric was calculated on a gene-specific basis for peaks as follows:
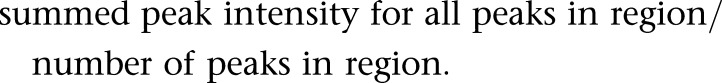


The set of formulas for defining multiple linear regression models are detailed below. For each species *i*:
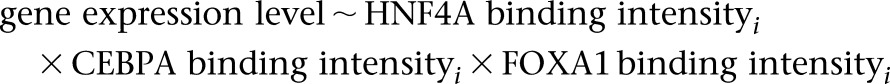

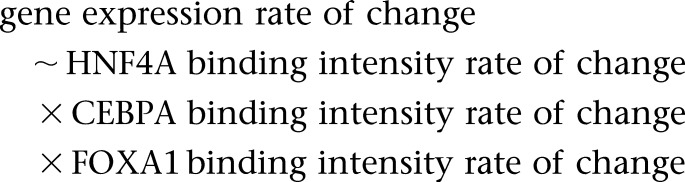

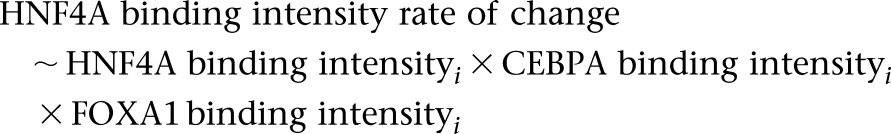

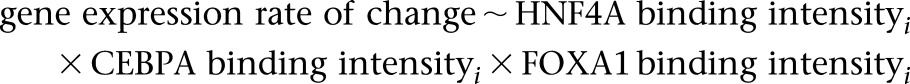


### Evolutionary rate comparison

We modeled the evolution of binding intensity and gene expression using a one-dimensional Brownian motion model with constant rate (Felsenstein 1985). The model simulates a stable continuous trait evolving under neutral drift with the degree of shared trait between species proportional to their shared ancestry. We defined the evolutionary tree as: (CAR: 3, (SPRET: 1.5, (BL6: 0.5, CAST: 0.5): 1): 1.5) (Dejager et al. 2009). The Brownian model, Ornstein-Uhlenbeck model, and maximum likelihood fitting procedure used are implemented in the R package Geiger (Harmon et al. 2008). Rates were log-transformed prior to analysis.

## Data access

All novel data sets from this study have been submitted to the ArrayExpress database (https://www.ebi.ac.uk/arrayexpress) under accession numbers E-MTAB-2483 and E-MTAB-2484. Processed data and R code can be found in the Supplemental Material and online at http://www.ebi.ac.uk/research/flicek/publications/FOG13/.

## Supplementary Material

Supplemental Material
