# Supplementary material for: Decoupling of evolutionary changes in transcription factor binding and gene expression in mammals
Source: Genome Res. 2015 Feb;25(2):167–78. doi: 10.1101/gr.177840.114 (PMC4315291; doi:10.1101/gr.177840.114)
Supplement: Supplemental Material [file supp_25_2_167__index.html]

Decoupling of evolutionary changes in transcription factor binding and gene expression in mammals — Decoupling of evolutionary changes in transcription factor binding and gene expression in mammals — Supplemental Material 

# Decoupling of evolutionary changes in transcription factor binding and gene expression in mammals

## Supplemental Material

**Files in this Data Supplement:**

- Supplemental Data.xlsx
- Supplemental Material.docx
