# Supplementary material for: Decoupling of evolutionary changes in transcription factor binding and gene expression in mammals
Source: Genome Res. 2015 Feb;25(2):167–78. doi: 10.1101/gr.177840.114 (PMC4315291; doi:10.1101/gr.177840.114)
Supplement: Supplemental Material [file supp_gr.177840.114_Supplemental_Material.docx]

**Supplementary materials**

**Decoupling of evolutionary changes in transcription factor binding and gene expression in mammals**

Emily S. Wong^1^, David Thybert^1^, Bianca Schmitt^2^, Klara Stefflova^2^, Duncan T. Odom^*2,3^ Paul Flicek^*1,3^

^1^ European Molecular Biology Laboratory, European Bioinformatics Institute, Wellcome Trust Genome Campus, Hinxton, Cambridge, CB10 1SD, UK.

^2^ University of Cambridge, Cancer Research UK - Cambridge Institute, Li Ka Shing Centre, Cambridge, CB2 0RE, UK.

^3^ Wellcome Trust Sanger Institute, Wellcome Trust Genome Campus, Hinxton, Cambridge, CB10 1SA, UK.

|  | Linear weighting | | | No distance weighting | | | Exponential d_0_=500 | | | Exponential d_0_=5000 | | |
| --- | --- | --- | --- | --- | --- | --- | --- | --- | --- | --- | --- | --- |
|  | p | dep | indep | p | dep | indep | p | dep | indep | p | dep | indep |
| HNF | 6.5e-5 | -0.014 | -0.10 | 2.4e-4 | -0.055 | -0.084 | 4.3e-5 | -0.023 | -0.13 | 4.9e-4 | -0.037 | -0.088 |
| CEB | 1.5e-3 | -0.044 | -0.068 | 1.9e-4 | -0.032 | -0.078 | 3.5e-4 | -0.032 | -0.072 | 4.2e-4 | -0.029 | -0.076 |
| FOX | 2.9e-4 | -0.67 | -0.83 | 0.7 | -0.88 | -0.81 | 2.3e-3 | -0.085 | -0.099 | 0.08 | -0.065 | -0.084 |

**Table S1**. **Values of slopes and p-values for linear regression based on different methods for integrating multiple TF binding sites to genes.**

Slopes were derived from the linear model: pairwise species binding intensity correlation coefficient ~ divergence time + ε, for each TF, where ε is the error variable. P-values from ANCOVAs were reported. These tested whether or not regression lines for TF dependent and independent genes were indistinguishable from one another. Peaks situated 10kb to either side of the TSS of protein-coding genes were used for analyses.

|  |  | OR | P-value | OR | P-value | OR | P-value |
| --- | --- | --- | --- | --- | --- | --- | --- |
|  |  | HNF4A | HNF4A | CEBPA | CEBPA | FOXA1 | FOXA1 |
| 10kb | BL6 | 1.73 | *2.2e-16* | 0.88 | 0.38 | 1.37 | 0.11 |
|  | CAR | 1.81 | *2.7e-11* | 1.10 | 0.55 | 1.93 | 0.01 |

**Table S2. Individual peaks between BL6 and CAR were more conserved for HNF4A dependent genes**

Pearson’s Chi-squared tests were used to assess whether individual peak locations were more conserved near TF dependent or TF independent genes at different distances from the TSS (10kb). Tests were performed against the background numbers of peaks in both species. Odds ratio and p-values for Pearson’s Chi-squared test with 1 degree of freedom are shown. P-values less than 1e-3 are shown in italics.

|  |  | OR | P-value | OR | P-value | OR | P-value |
| --- | --- | --- | --- | --- | --- | --- | --- |
|  |  | HNF4A | HNF4A | CEBPA | CEBPA | FOXA1 | FOXA1 |
| 10kb | CAST | 1.32 | *1.5e-6* | 0.92 | 0.59 | 0.87 | 0.49 |
|  | SPRET | 1.50 | *1.2e-6* | 1.42 | 0.02 | 1.51 | 0.06 |

**Table S3. Individual peaks between CAST and SPRET are better conserved at HNF4A dependent genes**

Pearson’s Chi-squared tests were used to assess whether individual peak locations were more conserved near TF dependent or TF independent genes at different distances from the TSS (10kb). Tests were performed against the background numbers of peaks in both species. Odds ratio and p-values for Pearson’s Chi-squared test with 1 degree of freedom are shown. P-values less than 1e-3 are shown in italics.

|  | HNF4A | CEBPA | FOXA1 |
| --- | --- | --- | --- |
| TF dependent | 0.74 | 0.70 | 0.67 |
| TF independent | 0.82 | 0.83 | 0.83 |

**Table S4. Preferential transcriptional divergence of TF dependent genes is also evident in mouse (BL6) versus rat comparison**

Using mouse and rat *de novo* assembled transcriptomic datasets (FPKM ­(fragments per kilobase of exon per million fragments mapped) values) from Kutter et al. 2012, we found similar relationship in gene expression divergence between TF dependent and TF independent genes. Values were log-transformed and a pseudocount of 1e-4 was added prior to analysis. Spearman’s rho values are presented in the table between mouse and rat comparisons. Correlation coefficients were consistently higher for TF independent genes in all cases consistent with the results of this study.

**
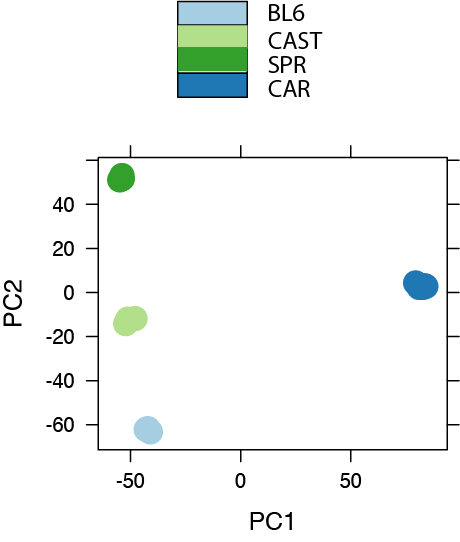
**

**Figure S1. Principle components analysis of read counts from expression datasets shows samples cluster by species**

Principle components analysis of the top 500 most variable genes across all expression datasets after normalization by library size and variance stabilizing transformation with the R package ‘DESeq’. The mean-dispersion relationship for each gene was estimated without information of species groupings prior to the transformation.

**
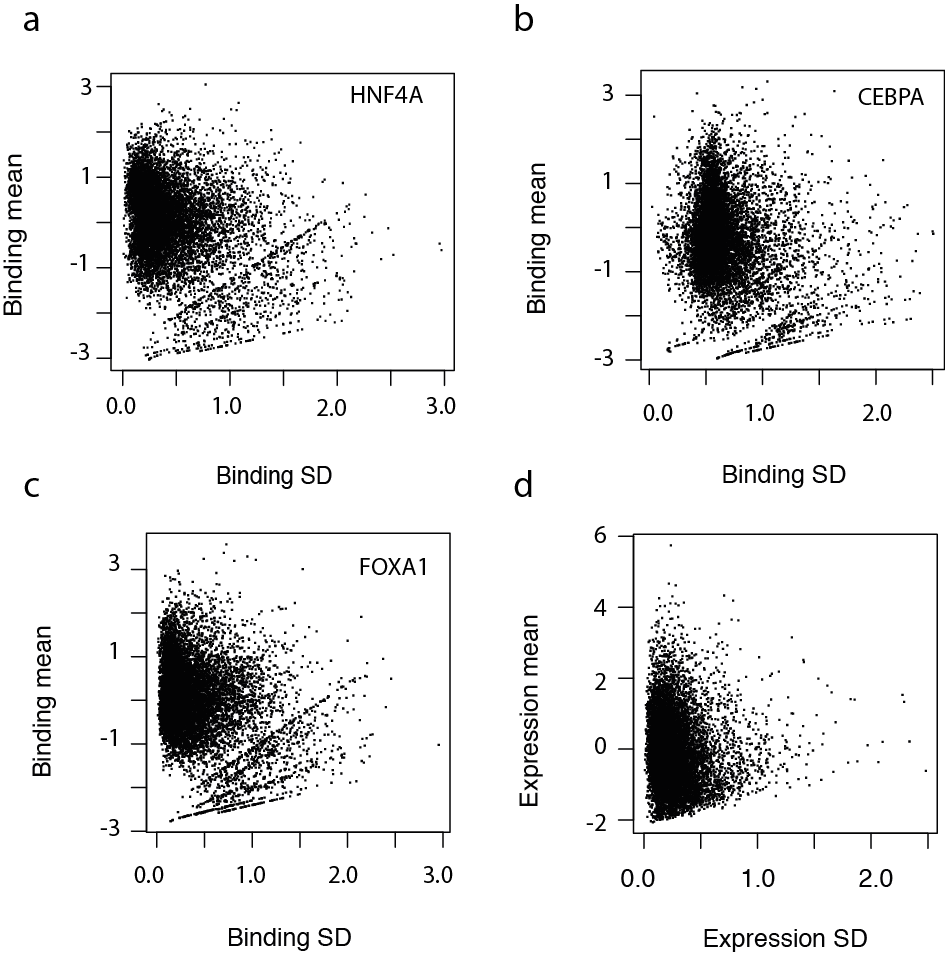
**

**Figure S2. Average binding intensities and gene expression level across species are negatively correlated with their standard deviation**

Log-transformed values across species were compared with their standard deviation.


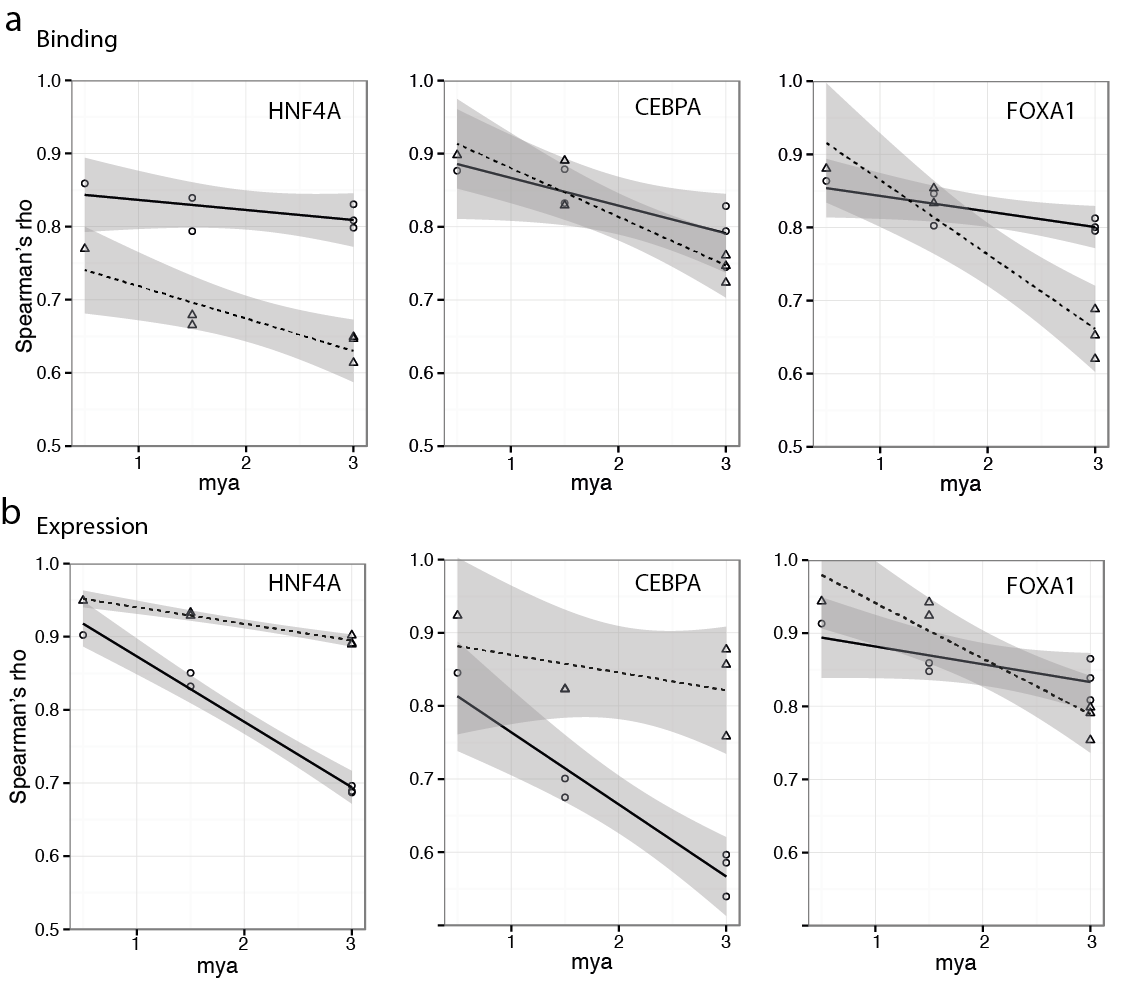


**Figure S3. Binding and expression changes in correlation coefficient for TF dependent versus subsampled TF independent genes against species divergence time**

**(A)** Changes in binding intensity correlation across species for TF independent subsampled genes (dashed line) and TF dependent genes (solid line) for each of the TFs. Shading denotes the point-wise 95% confidence interval for each group of genes.

**(B)** Changes in the correlation coefficient of gene expression across species for TF independent subsampled genes (dashed line) and TF dependent genes (solid line) for each of the TFs. Shading denotes the point-wise 95% confidence interval for each group of genes.


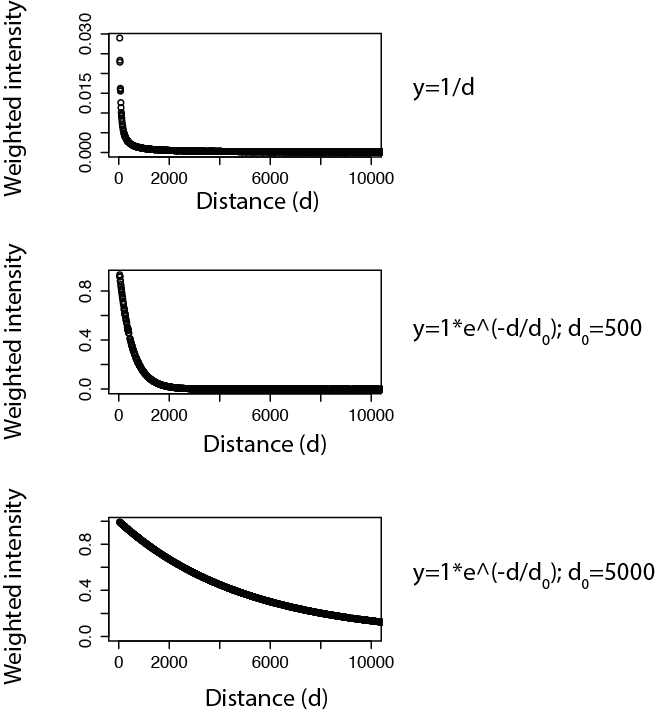


**Figure S4.** **Effect of different distance-based weighting schemes to peak intensity where distances were randomly sampled from a uniform distribution.**

The top plot illustrates the effect of the distance-based weighting scheme on peak intensity. This method was used throughout the manuscript. The bottom plots shows the methods tested where a constant, d_0_, of 500 and 5000 were used. A hypothetical peak intensity of one was used to create these plots.


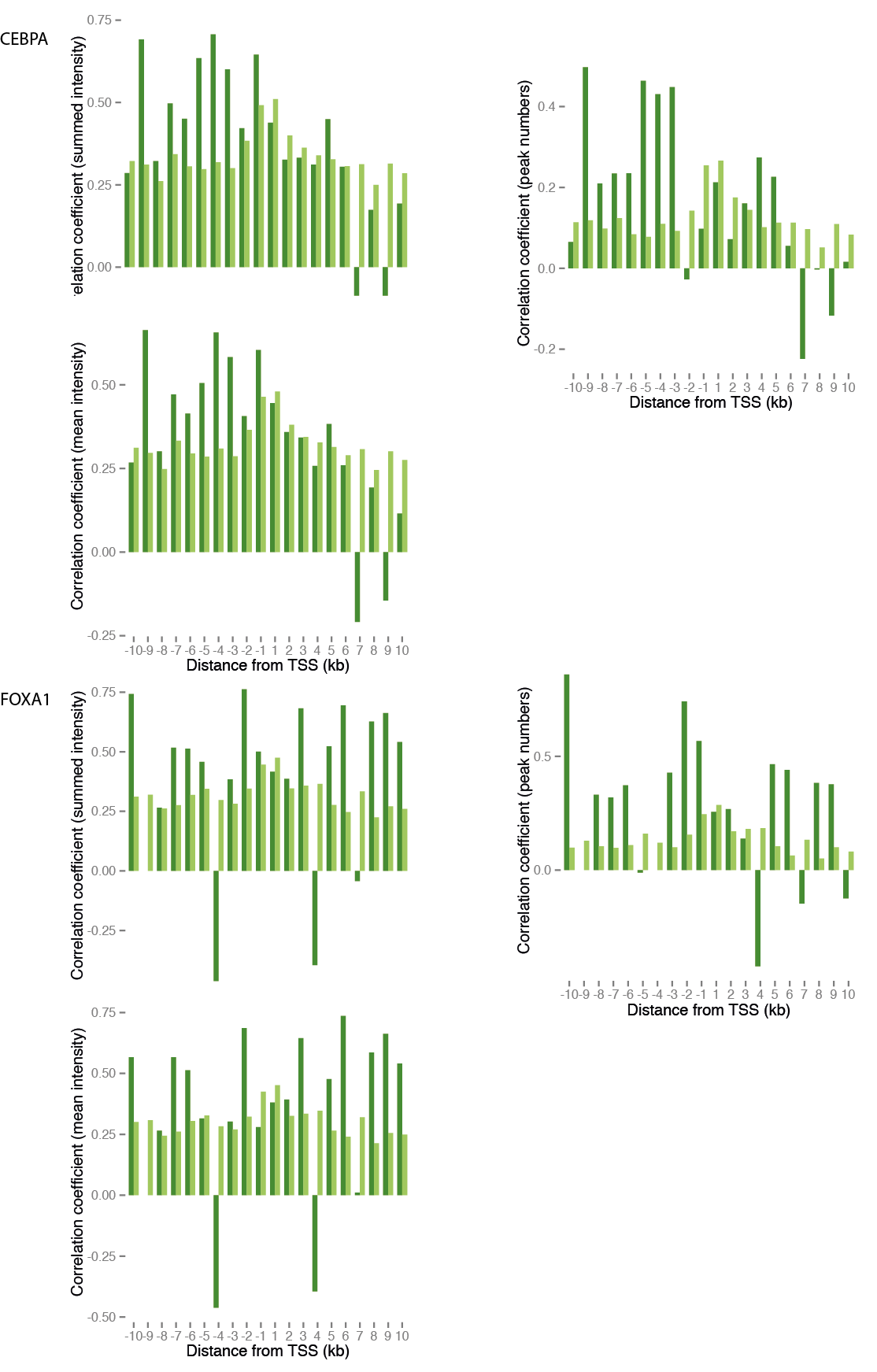


**Figure S5.** **Both peak intensity and total number of peaks are conserved near TF dependent genes.**

Spearman’s rho correlation coefficient of summed binding intensities (summation of all peak intensities within binned region), peak counts, average peak intensities (summed peak intensities divided by the number of peaks in each bin) averaged over BL6 and CAR, CAST and CAR and SPRET and CAR comparisons. These values are summarized for 1kb binned distances from TSS for TF dependent versus TF independent genes for all three TFs. HNF4A dependent (dark green) and HNF4A independent (light green).

Code snippets for main analyses outlined in the manuscript

# ---normalization of expression and binding intensities values where df is a data frame where each row denotes one expressed gene and bmean, castmean, smean and carmean are average expression values for BL6, CAST, SPR and CAR, respectively, across biological replicates

# expression values centre on mean adjust SD to 1

df$logbmean = log(df$bmean + 0.0001)

df$logcastmean = log(df$castmean + 0.0001)

df$logsmean = log(df$smean + 0.0001)

df$logcarmean = log(df$carmean + 0.0001)

df$logbmean = df$logbmean - mean(df$logbmean)

df$logbmean = df$logbmean/sd(df$logbmean)

df$logcastmean = df$logcastmean - mean(df$logcastmean)

df$logcastmean = df$logcastmean/sd(df$logcastmean)

df$logsmean = df$logsmean - mean(df$logsmean)

df$logsmean = df$logsmean/sd(df$logsmean)

df$logcarmean = df$logcarmean - mean(df$logcarmean)

df$logcarmean = df$logcarmean/sd(df$logcarmean)

# binding

df$logbbind = log(df$V1 + 0.0001)

df$logcastbind = log(df$V2 + 0.0001)

df$logsbind = log(df$V3 + 0.0001)

df$logcarbind = log(df$V4 + 0.0001)

df$logbbind = df$logbbind - mean(df$logbbind)

df$logbbind = df$logbbind/sd(df$logbbind)

df$logcastbind = df$logcastbind - mean(df$logcastbind)

df$logcastbind = df$logcastbind/sd(df$logcastbind)

df$logsbind = df$logsbind - mean(df$logsbind)

df$logsbind = df$logsbind/sd(df$logsbind)

df$logcarbind = df$logcarbind - mean(df$logcarbind)

df$logcarbind = df$logcarbind/sd(df$logcarbind)

# ---Brownian motion evolutionary rate analysis

# example of evolutionary rate estimation for HNF4a binding df is HNF4A dataframe of normalized binding intensities across all species and associated gene expression levels

library("geiger")

runfit = function( df, colnum ) {

res=data.frame(sel = numeric(),rate=numeric(), lnl=numeric())

for (i in 1:nrow(df)) {

a = (df[ i , c("logbmean", "logbbind")])

colnames(a) <- c("exp", "bind")

b = (df[ i , c("logcastmean", "logcastbind")])

colnames(b) <- c("exp", "bind")

c = (df[ i , c("logsmean", "logsbind")])

colnames(c) <- c("exp", "bind")

d = (df[ i , c("logcarmean", "logcarbind")])

colnames(d) <- c("exp", "bind")

x = do.call(rbind, list(a, b,c,d ))

rownames(x) = c("b","cast","s","car")

input = x[, colnum]

names(input)<- row.names(x)

bmfit = fitContinuous( tree, input, model="BM" )

res = rbind(res, c( bmfit$opt$sigsq, bmfit$opt$lnL, bmfit$opt$aicc))

}

return(res)

}

hnf_binding = runfit(df, 2)

#----explicit definition of how correlation coefficients for TF dependent genes and TF independent genes were calculated

# example with HNF4a data

target = subset( df, Row.names %in% hnf4atargets)

non_target = subset( df, ! Row.names %in% hnf4atargets)

# binding

hnf4a_target_cor = c(

cor.test( target$logbbind , target$logcastbind, method='spearman'),

cor.test( target$logbbind , target$logsbind, method='spearman'),

cor.test( target$logbbind , target$logcarbind, method='spearman'),

cor.test( target$logcastbind , target$logsbind, method='spearman'),

cor.test( target$logcastbind , target$logcarbind, method='spearman'),

cor.test( target$logcarbind , target$logsbind, method='spearman')

)

hnf4a_nontarget_cor = c(

cor.test( non_target$logbbind , non_target$logcastbind, method='spearman'),

cor.test( non_target$logbbind , non_target$logsbind, method='spearman'),

cor.test( non_target$logbbind , non_target$logcarbind, method='spearman'),

cor.test( non_target$logcastbind , non_target$logsbind, method='spearman'),

cor.test( non_target$logcastbind , non_target$logcarbind, method='spearman'),

cor.test( non_target$logcarbind , non_target$logsbind, method='spearman')

)

# -----Example: to test for differences between the trend in correlation coefficients in binding intensities between foxa1 dependent versus independent genes

mya = c(.5,1.5,3,1.5,3,3)

fox_bind = decaydf[,c("mya","fox_target_cor","fox_nontarget_cor")]

fox_bind = melt ( fox_bind, id="mya")

summary(lm( value ~ variable + mya, fox_bind))

a=aov( value ~ variable * mya, fox_bind)

b=aov( value ~ variable + mya, fox_bind)

anova(a,b)
